# Reconstructing of Embedded High-Aspect-Ratio Nano-Voids Generated by Ultrafast Laser Bessel Beams

**DOI:** 10.3390/mi11070671

**Published:** 2020-07-10

**Authors:** Tianqu Chen, Guodong Zhang, Yishan Wang, Xuelong Li, Razvan Stoian, Guanghua Cheng

**Affiliations:** 1 State Key Laboratory of Transient Optics and Photonics, Xi’an Institute of Optics and Precision Mechanics of CAS, Xi’an 710119, China; chentianqu17@mails.ucas.ac.cn (T.C.); yshwang@opt.ac.cn (Y.W.); 2 University of Chinese Academy of Sciences, Beijing 100049, China; 3 Electronic Information College, and Center of Optical Imagery Analysis and Learning, Northwestern Polytechnical University, Xi’an 710072, China; guodongzhang@nwpu.edu.cn (G.Z.); li@nwpu.edu.cn (X.L.); 4 Laboratoire Hubert Curien, UMR CNRS 5516, Université de Lyon, Université Jean Monnet, 42000 Saint Etienne, France; razvan.stoian@univ-st-etienne.fr

**Keywords:** bessel beam, femtosecond laser, picosecond laser, fused silica, micro-nano hole, high-aspect-ratio, tomography

## Abstract

Ultrafast non-diffractive Bessel laser beams provide strong light confinement and show robust advantages for fabricating high-aspect-ratio nanoscale structures inside transparent materials. They take the form of nanoscale voids with typical diameters well below the wavelength and aspect ratio of more than 1000. Delivering 3D morphologies of such nanoscale voids is an important issue to evaluate the result for fabrication. However, the characterization of such laser-induced structures is a difficult task. Here, an accurate and time-saving tomography-like methodology is proposed and adopted for reconstructing the morphology of high-aspect-ratio nano-holes. The technique allows an accurate assertion of laser parameters and position on nano-structured features. The reconstructed configuration reveals that nanoholes morphologies have a close relationship with energy distribution in the focal region. It suggests that the configuration of micro-explosion can be controlled by laser energy deposition in the process of laser-matter interaction down to the nanoscale.

## 1. Introduction

Bessel beam with an intrinsic non-diffracting character contains an intense central core that stays invariant over a long propagation distance defined by the characteristics of the focusing optics, outpacing the Gaussian confocal distance in similar conditions [1,2,3,4,5]. The core is surrounded by a series of successive rings defined by the interference orders of intersecting conical wavefronts. When encountering an obstacle, the beam can reconstruct itself using the rings reservoir. It thus exhibits high robustness during propagation [6,7]. These features make Bessel beams attractive in the field of laser machining. Here specifically, ultra-high aspect ratio (depth/diameter) nanovoids exceeding 1000:1 have been reported using ultrafast micro-Bessel beams [8,9,10]. They result from a strong energy concentration due to Bessel exposure, where the relaxation direction occurs perpendicular to the axis and results in the formation of nano-voids. This structuring process is remarkable by its dimensions and thus new approaches for the conception of the 3D photonic crystals, phonon crystals, metamaterials, integrated photonics devices, beam welding, and plasmonic devices could rely on this unique fabrication technique [11,12,13,14,15]. Typically, these embedded holes have taper-free profiles, hundreds of nanometers in diameter and a few hundreds of micrometers in-depth, as reported for example in fused silica and sapphire [10,16]. Notwithstanding the effectiveness of short pulse duration Gaussian-Bessel beams for drilling high aspect ratio channels, it is also possible to precisely manipulate the characteristics of the fabricated holes and to access diverse aspect ratios by regulating the beam parameters [17,18,19] or by phase engineering [20,21]. As a prerequisite for optimizing the structure and the fabrication process, the configuration of the nanohole should be obtained quickly and precisely.

However, despite the interest, it is yet a challenge to obtain full information of the nano-holes topographies. Optical microscopy including trans-illumination, phase contrast, and differential interference contrast (DIC) microscopy is an easy and common way to observe the 3D structures in transparent materials. In addition, the DIC and the phase contrast microscopy can effectively reveal the variation of the refractive index within the specimen, mapping optical phase variations into light amplitude variations [22]. The difference between optical images of the sample before and after water injection can indicate the existence of an embedded channel [8]. Nevertheless optical microscopy suffers from resolution limits and the accuracy of spatial information stays comparable to the half of wavelength of the illumination light. Therefore, the diameter information and the entire configuration of the nano-holes cannot be obtained with sufficient precision. Scanning electron microscope (SEM) can reveal morphologies with nanometer resolution, therefore, is appropriate for characterizing the cross-sections (perpendicular to the laser propagation direction) of the nano-holes. However, they only allow us to explore surface information. In order to get the 3D information of the nano-holes, for example, sectional and transverse information, SEM, should be utilized combined with the focused ion beam milling [22,23], which allows us to remove the surface material layer-by-layer and it is a time-consuming process. For these reasons, an effective and time-saving characterization method is necessary for optimizing the drilling processes and searching for the responsible mechanism.

In this letter, a time-saving tomography-like investigation of high-aspect-ratio nano-void configuration is proposed which only needs one-time SEM observation. The constructed image reveals that the cross-section of a nanohole typically consists of a central hollow void, circumjacent modified regions, and randomly distributed cracks outside the laser-irradiated region. Using this method, the dependence of the nano-hole configuration (both inside the material and on the back surface) on pulse duration and energy of a single pulse has also been investigated.

## 2. Experimental Setup

### 2.1. Materials

Polished parallelepiped fused silica block (Corning 7980, Shanghai, China) with a size of 20 mm × 10 mm × 4 mm was used as the sample in our experiments. The surface of the glass sample was cleaned ultrasonically (PS-20, Jeken, Dongguan, China) with ethanol before laser treatment.

### 2.2. Preparation of the High-Aspect-Ratio Nano-Holes with Bessel Beams

**Figure 1:**
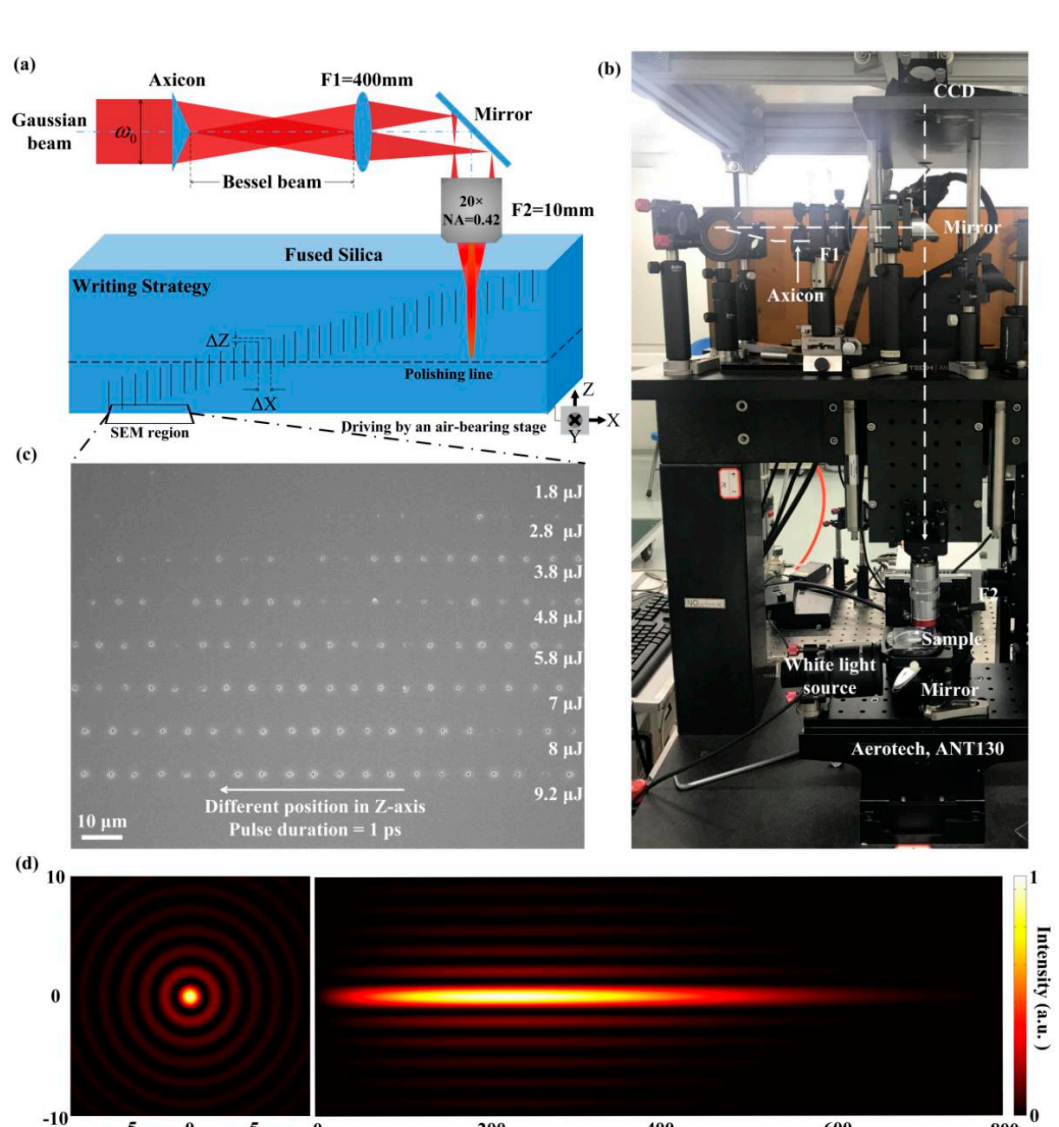
(**a**) Schematic setup for generating the tomography of high-aspect-ratio nano-hole. The dashed line indicates the polishing plane where the cross-section of nanohole is checked with SEM. (**b**) Setup of the Gaussian-Bessel pulse beam processing system. (**c**) Nano-holes array induced on the bottom surface of the fused silica which was revealed by SEM before polishing procedure. (**d**) Simulated linear intensity profiles of the Bessel beam inside the fused silica on axial and radial sections.

The experiment is performed with an Yb:KGW femtosecond laser (Pharos, Light conversion) which has a central wavelength of 1030 nm. The schematic of the beam shaping setup and exposure geometry is shown in Figure 1a. For a one-time tomography scan, the laser was set to 1 kHz and the irradiation was synchronized with the sample movement. The displacement between two adjacent positions is set to be Δx = 6 μm in x-direction and Δz = 0.9 μm in the z-direction. All the exposure is conducted by sequential single Bessel-Gaussian beam pulses, where the variety of the pulse durations from 250 fs to 6 ps acts as adjusting parameter. No observable inter-influence happened between two adjacent structures.

Figure 1b shows the Gaussian-Bessel pulse beam processing system used in the experiment. The setup basically consists of an axicon with a base angle of 1° to provide a first Bessel region, and a demagnifying 4f optical system (with a demagnifying factor of 40) made of a lens (f1 = 400 mm) and a microscope objective (20×, NA = 0.4, f2 = 10 mm, Mitutoyo NIR, working distance 20 mm). The 4f setup projects the Bessel beam to the sample to obtain a second Bessel region adapted for writing [24,25]. The pulse duration varies from 0.25 ps to 6 ps. The Gaussian pulse beams have an initial diameter of 7.6 mm. The simulated linear intensity profiles of the Bessel beam inside the fused silica on axial and radial sections as shown in Figure 1d. The cone angle of the second Bessel region in the fused silica is 12° and the central lobe of the Bessel beam is around 1.2 μm in diameter FWHM. The specimen is fixed on a high-precision air-bearing stage (ANT130, Aerotech, Shanghai, China), which allows the sample to be moved with high precision relative to the position of the laser beam during laser processing. The entire process is monitored by a CCD camera. The bulk exposure ensured embedded structures for characterization where the relaxation of energy occured were completely under confinement.

Besides the characterization of embedded structures, we are equally interested in a hole morphology intersecting the surface and, by this, providing a way to relax the inner pressure by the valve-like opening. Figure 1c shows the nano-holes array on the back surface of the fused silica which was revealed by SEM before the polishing procedure.

During the procedure besides the pulse duration, various single pulse energies from 0.8 μJ to 9.2 μJ were employed to study the effect of Bessel beam energy on the morphology of the nano-structure. This duration/energy combination defines the perimeter of a large landscape of processing conditions.

### 2.3. Measurement and Characterization

After polishing step the back surface of the sample was characterized using field emission scanning electron microscopy (SEM, JSM-7500F, JEOL Ltd., Co., Tokyo, Japan). Each time before SEM, an analysis ultrasound bath with distilled water was used in order to keep the sample clean. Thus the structures are revealed gradually, constructing their 3D profiles.

Compared to the focused Gaussian beam whose aberration is demonstrated to be strongly related with the focus depth in bulk material causing laser-modification to be inconstant [26], the demagnified Bessel beam in the experiment, with a thin toroid on the entrance of objective, is observed to have negligible aberration fluctuation within millimeter range during focus moving along the z-axis. The Bessel beam-induced nano-holes with focus position varying in millimeter range in z-axis can be approximately considered as unaffected by spherical aberration. Previous results showed that Bessel processing possesses excellent repeatability [27,28,29]. In a region of several of millimeters on the x-axis, given the repeatability of the ultrashort pulse structuring, we assume that all the nanoholes are actual replicas of the central one. The different positions of adjacent holes in z-direction allow us to estimate that every SEM image shows the structural morphology of the same nanohole at different depth level. Relative position information of two observation points can be accurately measured and stored by the location recording function of the SEM system. Thus, the depth information of the hole can be calculated based on the geometric relationship shown in Figure 1a which is *h* = l × Δ*z*/Δ*x*, where *l* is the distance on the x-axis between the first and the last hole and *h* represents the depth of the hole. With the diameter and depth information, we obtained the profile of high-aspect-ratio nano-hole drilled with ultrafast Bessel beam can be reconstructed with only one-time SEM analysis. We believe this is a much more efficient way to analyze the inner structure of nano-holes with relative hundreds of micrometer-length on z-axis than the traditional characterization methods we mentioned above.

After the exposure with ultrafast laser beams, mechanical milling has been used for revealing the inside cross-section of the nano-hole (MPD-1, MOWEI, Shanghai, China). With the help of optical microscopy, polishing depth can be determined with an accuracy of several micrometers. The mechanically polishing procedure used 1 μm roughness of cerium oxide polishing powder. After polishing procedure, quick treatment with a low concentration of hydrofluoric acid (soaking in 5% hydrofluoric acid for 30 s at room temperature) and an ultrasound bath with distilled water and ethanol have been used in order to remove surface impurities.

## 3. Reconstruction and Effect of Pulse Duration on the Inside Nano-Structure

Non-diffractive beams can potentially determine a material response that can bypass optical limits and achieve structural features not limited by optical resolution [6]. The void-like nano-structure induced by a single-pulse Bessel beam in the fused silica is highly dependent on the laser parameter in terms of spatial extent. The principal goal of this work is to reconstruct the internal structure of the nano-hole and reveal the influences of Bessel beam processing of fused silica.

Pulse duration is a key factor to control the configuration of the nanohole induced by ultrafast Bessel-Gaussian beam and the reason is related to the efficiency of energy deposition. The laser pulse of short temporal duration (sub-ps) can trigger significant multiphoton ionization, generating free electrons at the beginning of the pulse, which would ultimately defocus the rest of the laser pulse, decreasing the incoming laser energy density along the axis [30,31,32,33]. The result is mostly a small refractive index increase. Nonlinear propagation models together with experimental results showed that a laser pulse of picosecond pulse length allows for a more efficient deposition of the laser energy into the sample relying on the dynamics of carrier generation and their light scattering efficiency. Electronic behavior and excitation profiles allow us to counteract defocusing and concentrate energy. Void-like regions can be obtained by what is standardly called a microexplosion [34], implying transitions to dense plasma states, phase transitions, and mechanical rarefaction and decomposition [33,35]. The scenario was verified in bulk fused silica and one example is discussed below.

**Figure 2:**
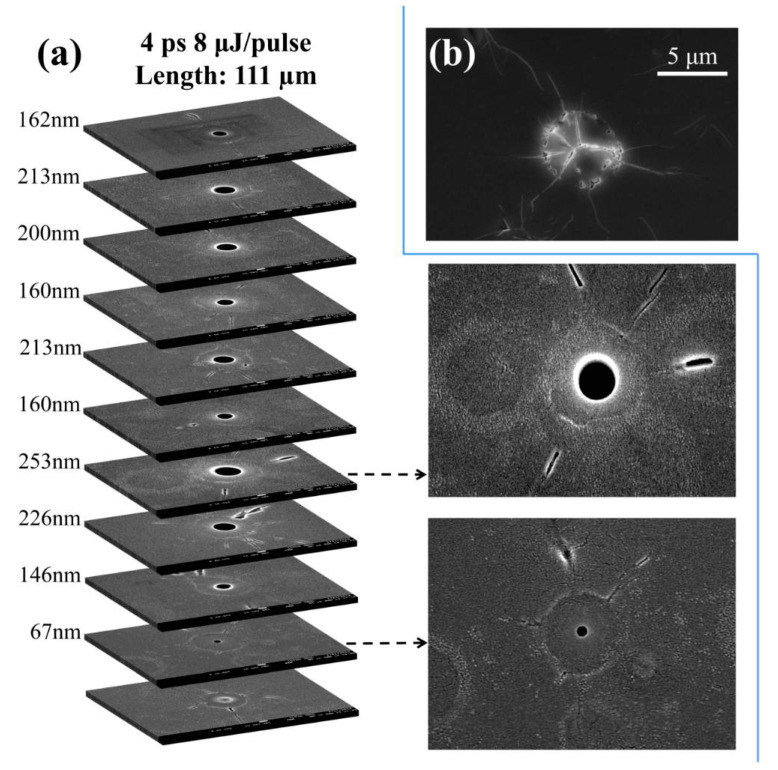
(**a**) Tomography of hole drilling by a single ultrafast Bessel beam pulse. Pulse energy is 8 μJ with 4 ps pulse duration. (**b**) Radial configuration of structure induced by 150 fs Gaussian beam pulses.

**Figure 3:**
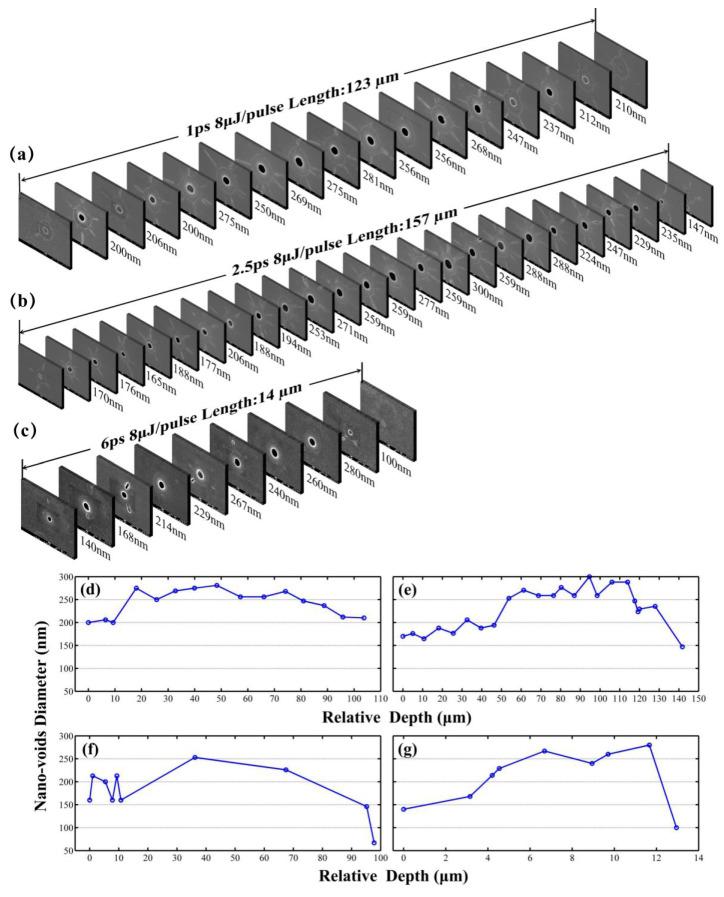
Tomography of the hole drilled by a single ultrafast Bessel beam pulse. Pulse energy keeps constant at 8 μJ/pulse. Different pulse durations are used, shown in (**a**–**c**). Corresponding curves of nano-holes diameter and relative depth are given above, where (**d**–**g**) represent curves for 1 ps, 2.5 ps, 4 ps and 6 ps cases respectively. Take the position of the first hole as zero relative depth.

After the polishing procedure, nano-holes induced by long pulse width beams can be revealed, as shown in Figure 2 and Figure 3. Figure 2a shows a series of cross-sections of a nanohole inside fused silica fabricated with a pulse duration of 4 ps and an energy of 8 μJ in Bessel beam configuration. The maximum diameter is 253 nm, and the length of nanohole is about 111 μm.

The insets in Figure 2a shows two magnified images corresponding to the middle and bottom of the nanohole. The diameters of cross-section indicate there is a relationship between the diameter of nanovoid and laser peak intensity in the focal region. The structures shown in the insets consists of three parts: central hollow void, circumjacent modified region, and randomly distributed cracks outside the laser-irradiated region. The circularly-modified region plausibly comes from the polling due to thermal wave diffusion and subsequent modification of the silica matrix [35,36]. This region is spatially different from the extent of the ring of the Bessel beam since a similar structure has also been observed with Gaussian beam writing, shown in Figure 2b. The ring size in the bottom picture is smaller than in the middle picture and the radius of the modification region is dependent on source energy density. These randomly distributed cracks seem to be not related to the laser polarization. It is attributed to the stress release after laser irradiation [31,37].

Nonlinear processes involving the formation of nanoholes are sensitive to the pulse duration of the exposure laser. A series of experiments have been conducted to investigate the configurations of nanoholes under pulse durations varying from 0.25 ps to 6.0 ps. Figure 3 represents the configurations of structures written with different pulse durations inside fused silica. We take the position of the first hole as a zero relative depth point and the general profile of nano-hole can be revealed by the diameter curve. The relative position on the z-axis of each nano-hole can be read from Figure 3d–g. Compared to the focal length of the Bessel beam (about 600 μm, shown in Figure 1d), the length of nano-hole was much smaller which indicates the area over the threshold was concentrated in the central focal region. Since the relative depth of the nano-hole was less than 160 μm, the length of the observation region on z-axis only needed to be less than 200 μm. There are three kinds of damage induced by a single ultrafast laser pulse in fused silica. For the pulse duration of 250 fs and 500 fs, weak exposure generates transient small density plasma and a very weak structural change in the focal region. Here, after the relaxation of the glass, the matrix recovers without a measurable refractive index change checked by phase-contrast microscopy. This kind of exposure can be used to eliminate the nanoscale structural defects under the surface in optical glass [38]. When the pulse duration increases to 1 ps the nanohole become visible under SEM. The 2.5 ps pulse reaches the maximum length and the corresponding area of cracks is also maximum. It can be explained as the a longer temporal duration laser leading to a more efficient energy deposition, while the relatively low efficiency of plasma defocusing allows concentrating axial energy, resulting in a strong plasma and high electronic density [35]. This represents an optimum in energy deposition where light confinement balances smaller photoionization cross-section. The peak intensity in the focal region decreases for the cases of 4 ps and 6 ps laser pulse. In particular, the length induced by a 6 ps decreases dramatically in comparison to that of 4 ps pulse. It suggests that the energy deposit is nonlinear, and the formation mechanism of nanohole is highly nonlinear too. The further increase of pulse duration significantly limits the excitation effectiveness. These observations underline the efficiency of the proposed observation method for aberration-free observation micro-region.

**Figure 4:**
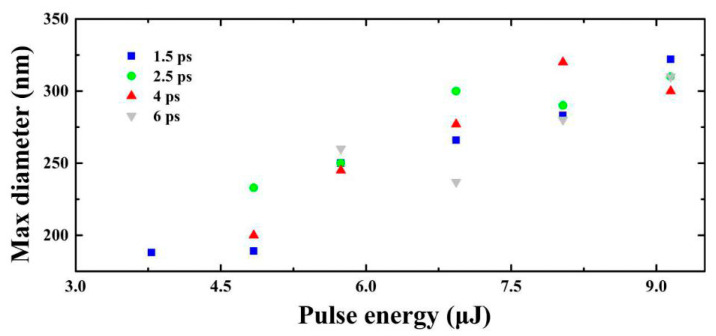
Maximum diameters of inside holes as a function of pulse energy measured inside the fused silica for different pulse widths.

The maximum diameters of these internal nano-holes induced with different parameters have been concluded based on the experimental measurements, shown in Figure 4. We find that the maximum diameter increases with the increase of the pulse energy. However, there is no straightforward relationship between the maximum diameter and the input energy due to the character of nonlinear pulse propagation that creates a complex dependence between input energy and axial energy density.

## 4. Discussion about Nano-Structure on Exit Surface

### 4.1. Effect of Pulse Energy and Pulse Width on Exit Surface Nano-Structure

**Figure 5:**
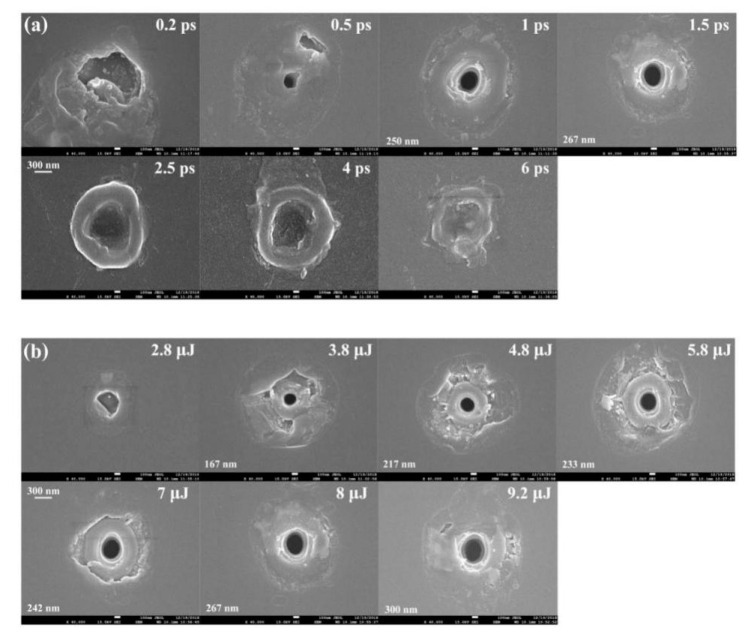
SEM characterization of nano-structure on the exit surface of the fused silica sample for different laser parameters. (**a**) Pulse width scan results at 8 μJ/pulse cases. (**b**) Energy scan results for a fixed pulse width of 1.5 ps. Diameters of the nano-holes have been marked in each figure. The relative spatial positions of the laser beam and the bottom surface are constant.

Figure 5 shows the geometrical characteristics of the nano-structure on the back exit surface of the fused silica sample. The track morphology depends significantly on the pulse duration and pulse energy. As we can see, the nano-hole structure can only be observed at 0.5–1.5 ps cases. The surface morphology shows a big difference between the case of pulse duration τ < 1 ps and the case τ > 1.5 ps, as shown in Figure 5a. For pulses higher than 2.5 ps, we can observe significant signs of melting on the exit surface [39].

For pulse durations between 2.5 ps and 6 ps, the material in the center of the laser irradiation area is melted by long pulse width laser pulses and transferred to liquid phase [36,40]. These materials flowed out the surface driven by the hydrodynamic movement and internal pressure. After cooling and re-solidification on the bottom surface, a crater-like structure formed in the irradiated region. Combining with the results shown in Figure 2 and Figure 3, we considered that the flowing out material, shown in Figure 5a, covered the hole-structure. The re-solidification material existed around the central area and the outline of the crater structure become unclear with pulse width increase.

According to the results of the energy scanning experiment shown in Figure 5b for a given pulse duration of 1.5 ps, the nano-hole structure appears first at 3.8 μJ/pulse case and the diameters of the nanoholes become larger (from 167 nm at 3.8 μJ/pulse case to 300 nm at 9.2 μJ/pulse) as the laser energy increases. Based on the diameter of the central lobe (1.2 μm for FWHM), the intensity is about F = 2.24 J/cm^2^ for 3.8 μJ/pulse case and F = 5.42 J/cm^2^ for 9.2 μJ/pulse case. Much more splashes around the hole indicating that the volume of material flowing out from the inside is larger.

**Figure 6:**
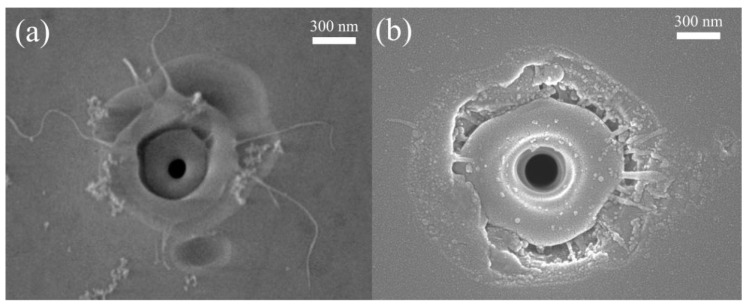
SEM images of the crater on the back surface of fused silica. The single pulse is (**a**) 3 μJ and (**b**) 8 μJ with a pulse duration of 1ps.

Figure 6a shows the configurations on the surface without cleaning after laser ablation. Nano-fiber remains on the surface in Figure 6a and shows the flow of liquid droplet and re-solidification of silica on the surface, similar to the other observations [41,42]. When the pulse energy goes to 8 μJ, Figure 6b, the signal of brittle fracture and deposition appear on the periphery of the crater. It is probable that low energy laser irradiation induces a flow of liquid phase or extending on a limited region, while intense laser irradiation generates a more energetic liquid extending further away. The expansion drive could indicate an internal pressure resulting from gas-phase nucleation. The granular remains on the surface of the crater seem to come from the deposition of the vapor phase [16].

**Figure 7:**
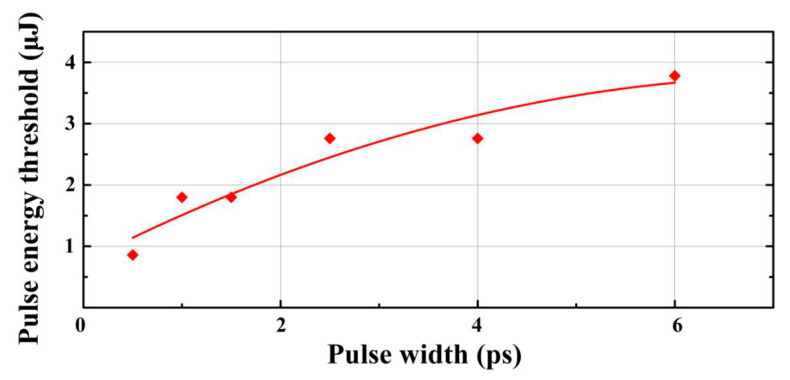
Bottom surface damage threshold versus different pulse duration. The line is added to guide the eye.

The bottom surface damage threshold of silica material can be obtained from the occurrence of the damage trace which can be observed by the SEM test, as shown in Figure 1c and Figure 7. In the case of a pulse width of 0.5 ps, only 0.9 μJ of energy is required to induce the surface damage, while for 6 ps case 3.8 μJ/pulse is needed. Based on the beam size given above, the local fluence for 0.5 ps and 6 ps cases are F_0.5_ = 0.53 J/cm^2^, F_6_ = 2.24 J/cm^2^ respectively. The bottom surface damage threshold in the case of Bessel beam processing seems to increase with the increase of pulse width and this phenomenon is consistent with the results of [43,44].

### 4.2. Effect of Relative Position of the Pulse Beams to the Exit Surface on Nano-Structure

Considering the Bessel beam has long nondiffracting length compared to the traditional Gaussian beam, they can be used for direct-write of feature in the bulk or on the surfaces or through drilling in transparent materials [27,45]. It is necessary and meaningful to study the effect of the relative position of the pulse Bessel beams to exit surface on nano-structure morphology and dimension [10].

**Figure 8:**
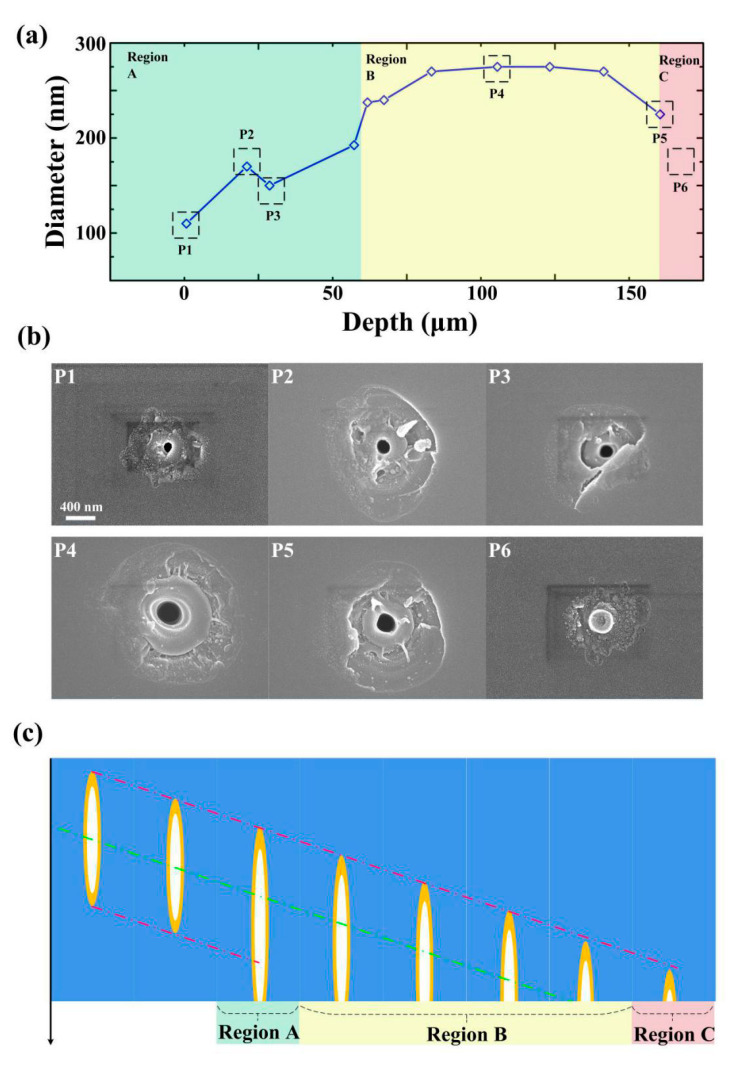
Effect of the relative position of the pulse beams to the exit surface on nano-structures (**a**) Nano-holes diameter corresponding to three different morphology-based region (uniquely colored zones) of Bessel beam irradiation on the exit surface of fused silica. (**b**) The nano-structures on the bottom surface which are induced by 1.5 ps, 8 μJ/pulse single pulse laser beams are presented corresponding to the different positions. (**c**) Schematic diagram of confined micro explosion and liquid expulsion taking place in Region A, Region B, and Region C. The dot green line shows the symmetry plane of the Bessel beam, located in the middle of the interference region. The red line represents the bottom of the laser-modified region, which surrounds the nano-hole.

When the focal plane of the Bessel beam was near the exit surface, the length of the nanohole increased to about 1.5 times than in bulk in the case of 1 ps and 8 μJ pulse energy. The diameters of craters on the exit surface also depend on the position of the focus. We sketch the change of the nanohole’s configuration with the movement of the focus position in the bulk sample. The diameter increases first and then decreases as the Bessel beam moves out of the bottom surface, shown in Figure 8a. Region A represents the area where the nano-hole structures were induced by a relaxation process quasi embedded. The diameter of nano-holes (110 nm) in this region was dramatically smaller than the other region. The first figure part in Region A reveals the obviously modified zone around the nano-hole and there were no splashes around. We consider that this nano-hole was induced by a confined relaxation entraining the softening area on the bottom surface. The mechanical spall conditions can be relaxed if the solid undergoes fast transitions to the soft or the liquid phase before the mechanical spall. As the beam continues to move to the surface, there are small amounts of splashes around the nano-hole combined with sign of brittle fracture and the diameter is still small, see P2 and P3 in Figure 8b.

Region B represents the area where the nano-hole is induced by the Bessel beam itself. The size of the hole in this region is much bigger (275 nm) and there were many more splashes around the hole, shown in P4 in Figure 8b. In the case of P5, the beam almost leaves the bottom surface. The hole size becomes small (225 nm) combined with fewer splashes. Region C means there is no hole-structure and the beam leaves the bottom surface. As shown in P6 in Figure 8b, the circumjacent modified region is similar to that observed in P1, while the central area has no hole-structure but thermal damage trace. This sign indicates that the surrounding area has been softened by a laser beam while there is no sufficient energy or internal jet formation for material rupture.

From Figure 3 and Figure 8a, we draw the conclusion that the three-dimensional configuration of the hole is decided by laser intensity distribution in the bulk sample. The Schematic diagram of confined micro explosion and jet formation that happened in Region A, Region B, and Region C are shown in Figure 8c. The theoretical spallation criterion in a solid requires that the elastic energy exceeds the cohesive energy and Young’s Modulus [46,47,48]. The softened glass has small Young modulus which makes the internal material easier to expel [49,50]. Combined with a discussion about the evacuation of liquid material above, we can draw the conclusion that the amount of splashed material correlates with the diameter and relates to the local energy density and liquid-phase-transformation plays an important role in the nanohole formation.

## 5. Conclusions

A convenient solution has been introduced for obtaining 3D tomography-like characterization of high-aspect-ratio nano-holes drilled with ultrafast Bessel beams. Extended information about the nano-holes has been revealed in detail with the help of this quick method. The dependence of the nano-holes morphologies on pulse duration and energy of single pulses have been investigated in the experiment. The effect of the relative position of the pulse beams to the bottom interface on the nano-structure has been discussed. Liquid expulsion has been observed which indicates liquid is involved in the formation process of nano-holes. The morphological characterization revealed several formation mechanisms of bottom surface nano-hole structure, which is of great significance to further reveal the laser-material-interaction mechanism on the interface. The reconstructing configuration reveals that the morphology of the nano-hole has a close relationship with energy distribution in the focal region. This optimization of laser parameters will improve the accuracy of manufacturing nano-structures and nano-devices in transparent dielectric materials.

## References

[1] Durnin J., Miceli J.J., Eberly J.H. (1987). Diffraction-free beams. Phys. Rev. Lett..

[2] Sheppard C.J.R., Wilson T. (1978). Gaussian-beam theory of lenses with annular aperture. J. Microw. Opt. Acoust..

[3] McLeod J.H. (1954). The axicon: A new type of optical element. J. Opt. Soc. Am..

[4] Herman R.M., Wiggins T.A. (1991). Production and uses of diffractionless beams. J. Opt. Soc. Am. A.

[5] Gori F., Guattari G., Padovani C. (1987). Bessel-Gauss beams. Opt. Commun..

[6] Stoian R., Bhuyan M.K., Rudenko A., Colombier J.P., Cheng G. (2019). High-resolution material structuring using ultrafast laser non-diffractive beams. J. Adv. Phys. X.

[7] Stoian R., Bhuyan M.K., Zhang G., Cheng G., Meyer R., Courvoisier F. (2018). Ultrafast Bessel beams: Advanced tools for laser materials processing. Adv. Opt. Technol..

[8] Bhuyan M.K., Velpula P.K., Colombier J., Olivier T., Faure N., Stoian R. (2014). Single-shot high aspect ratio bulk nanostructuring of fused silica using chirp-controlled ultrafast laser Bessel beams. Appl. Phys. Lett..

[9] Mitra S., Chanal M., Clady R., Mouskeftaras A., Grojo D. (2015). Millijoule femtosecond micro-Bessel beams for ultra-high aspect ratio machining. Appl. Opt..

[10] Meyer R., Froehly L., Giust R., Del Hoyo J., Furfaro L., Billet C., Courvoisier F. (2019). Extremely high-aspect-ratio ultrafast Bessel beam generation and stealth dicing of multi-millimeter thick glass. Appl. Phys. Lett..

[11] Zhang G., Stoian R., Zhao W., Cheng G. (2018). Femtosecond laser Bessel beam welding of transparent to non-transparent materials with large focal-position tolerant zone. Opt. Express.

[12] Jenne M., Flamm D., Ouaj T., Hellstern J., Grossmann D., Kumkar M., Nolte S. (2018). Glass cutting optimization with pump-probe microscopy and Bessel beam profiles. Proc. SPIE.

[13] Jenne M., Flamm D., Ouaj T., Hellsern J., Kleiner J., Grossmann D., Koschig M., Kaiser M., Kumkar M., Nolte S. (2018). High-quality tailored-edge cleaving using aberration-corrected Bessel-like beams. Opt. Lett..

[14] Courvoisier F., Couairon A., Stoian R. (2016). Ultrafast laser micro- and nano-processing with nondiffracting and curved beams. Opt. Laser Technol..

[15] Miyamoto I., Sugioka K., Sigmon T.W. (2000). Laser precision microfabrication. Laser Precis. Microfabr..

[16] Rapp L., Meyer R., Giust R., Furfaro L., Jacquot M., Lacourt P.A., Dudley J.M., Courvoisier F. (2016). High aspect ratio micro-explosions in the bulk of sapphire generated by femtosecond Bessel beams. Sci. Rep..

[17] Sudrie L., Couairon A., Franco M., Lamouroux B., Prade B., Tzortzakis S., Mysyrowicz A. (2002). Femtosecond Laser-Induced Damage and Filamentary Propagation in Fused Silica. Phys. Rev. Lett..

[18] Liu X., Sanner N.S., Sentis M., Stoian R., Zhao W., Cheng G., Utéza O. (2018). Front-surface fabrication of moderate aspect ratio micro-channels in fused silica by single picosecond Gaussian-Bessel laser pulse. Appl. Phys. A.

[19] Liu X., Li Q., Sikora A., Sentis M., Uteza A., Stoian R., Zhao W., Cheng G., Sanner N. (2019). Truncated Gaussian-Bessel beams for short-pulse processing of small-aspect-ratio micro-channels in dielectrics. Opt. Express.

[20] Meyer R., Jacquot M., Giust R., Safioui J., Rapp L., Furfaro L., Lacourt P.A., Dudley J.M., Courvoisier F. (2017). Single-shot ultrafast laser processing of high-aspect-ratio nanochannels using elliptical Bessel beams. Opt. Lett..

[21] He F., Yu J.J., Tan Y.X., Chu W., Zhou C.H., Cheng Y., Sugioka K. (2017). Tailoring femtosecond 1.5-μm Bessel beams for manufacturing high-aspect-ratio through-silicon vias OPEN. J. Sci. Rep..

[22] Zhang G., Cheng G., Bhuyan M.K., D’Amico C., Stoian R. (2019). Ultrashort Bessel beam photoinscription of Bragg Gratings waveguides and their application as temperature sensor. Photonics Res..

[23] Gamaly E.G., Juodkazis S., Nishimura K., Misawa H., Luther-Davies B., Hallo L., Nicolai P., Tikhonchuk T.V. (2006). Laser-matter interaction in the bulk of a transparent solid: Confined microexplosion and void formation. Phys. Rev. B Condens. Matter Mater. Phys..

[24] McGloin D., Dholakia K. (2005). Bessel beams: Diffraction in a new light. Contemp. Phys..

[25] Duocastella D., Arnold C.B. (2012). Bessel and annular beams for materials processing. Laser Photonics Rev..

[26] Mauclair C., Mermillod-Blondin A., Huot N., Audouard E., Stoian R. (2008). Ultrafast laser writing of homogeneous longitudinal waveguides in glasses using dynamic wavefront correction. Opt. Express.

[27] Bhuyan M.K., Courvoisier F., Lacourt P.A., Jacquot M., Dudley J.M. (2010). High aspect ratio taper-free microchannel fabrication using femtosecond Bessel beams. Opt. Express.

[28] Zhang G., Cheng G., Bhuyan M., D’Amico C., Stoian R. (2018). Efficient point-by-point Bragg gratings fabricated in embedded laser-written silica waveguides using ultrafast Bessel beams. Opt. Lett..

[29] Burakov I.M., Bulgakova N.M., Stoian R., Mermillod-Blondin A., Audouard E., Rosenfeld A., Husakou A., Hertel I.V. (2007). Spatial distribution of refractive index variations induced in bulk fused silica by single ultrashort and short laser pulses. J. Appl. Phys..

[30] Gadonas R., Jarutis V., Paškauskas R., Smilgevicius A., Stabinis A., Vaicaitis V. (2001). Self-action of Bessel beam in nonlinear medium. Opt. Commun..

[31] Faccio D., Rubino E., Lotti A., Couairon A., Tamosauskas G., Papazoglou D.G. (2012). Nonlinear light-matter interaction with femtosecond high-angle Bessel beams. Phys. Rev. A.

[32] Doring S., Szilagyi J., Richter S., Zimmermann F., Richardson M., Tünnermann A., Nolte S. (2012). Evolution of hole shape and size during short and ultrashort pulse laser deep drilling. Opt. Express.

[33] Polesana P., Franco M., Couairon A., Faccio D., Trapani P.D. (2008). Filamentation in Kerr media from pulsed Bessel beams. Phys. Rev. A.

[34] Juodkazis S., Nishimura K., Tanaka S., Misawa H., Gamaly E.G., Luther D.B., Hallo L., Nicolai P., Tikhonchuk V.T. (2006). Laser-induced microexplosion in the bulk of a sapphire crystal: Evidence of Mb pressures. Phys. Rev. Lett..

[35] Velpula P.K., Bhuyan M.K., Courvoisier F., Zhang H., Colombier J.P., Stoian R. (2016). Spatio-temporal dynamics in nondiffractive Bessel ultrafast laser nanoscale volume structuring. Laser Photonics Rev..

[36] Bhuyan M.K., Somayaji M., Mermillod-Blondin A., Bourquard F., Colombier J.P., Stoian R. (2017). Ultrafast laser nanostructuring in bulk silica, a “slow” microexplosion. Optica.

[37] Yu Y., Jiang L., Cao Q., Xia B., Wang Q., Lu Y. (2015). Pump-probe imaging of the fs-ps-ns dynamics during femtosecond laser Bessel beam drilling in PMMA. Opt. Express.

[38] Cao Q., Zhang J.J., Du J., Zhao H.M., Liu S., Peng Q. (2017). Athermal repair of nanoscale defects in optical materials using a femtosecond laser. Nanoscale.

[39] Kraus R.G., Stewart S.T., Swift D.C., Bolme C.A., Smith R.F., Hamel S., Hammel B.D., Spaulding D.K., Hicks D.G., Eggert J.H. (2012). Shock vaporization of silica and the thermodynamics of planetary impact events. J. Geophys. Res..

[40] Mermillod-Blondin A., Burakov I.M., Meshcheryakov Y.P., Bulgakova N.M., Audouard E., Rosenfeld A., Husakou A., Hertel I.V., Stoain R. (2008). Flipping the sign of refractive index changes in ultrafast and temporally shaped laser-irradiated borosilicate crown optical glass at high repetition rates. Phys. Rev. B.

[41] Venkatakrishnan K., Vipparty D., Tan B. (2011). Nanofibre fabrication by femtosecond laser ablation of silica glass. Opt. Express.

[42] Manickam S., Venkatakrishnan K., Tan B., Venkataramanan V. (2009). Study of silicon nanofibrous structure formed by femtosecond laser irradiation in air. Opt. Express.

[43] Stuart B.C., Feit M.D., Herman S., Rubenchik A.M., Shore B.W., Perry M.D. (1996). Optical ablation by high-power short-pulse lasers. J. Opt. Soc. Am. B.

[44] Perry M.D., Stuart B.C., Banks P.S., Feit M.D., Yanovsky V., Rubenchik A.M. (1999). Ultrashort-pulse laser machining of dielectric materials. J. Appl. Phys..

[45] Amako J., Sawaki D., Fujii E. (2003). Microstructuring transparent materials by use of nondiffracting ultrashort pulse beams generated by diffractive optics. J. Opt. Soc. Am. B.

[46] Wang J., Rajendran A.M., Dongare A.M. (2015). Atomic scale modeling of shock response of fused silica and a-quartz. J. Mater Sci..

[47] Grady D.E. (1988). The spall strength of condensed matter. J. Mech. Phys. Solids.

[48] Uteza O., Sanner N., Chimier B., Brocas A., Varkentina N., Sentis M., Lassonde P., Legare F., Kieffer J.C. (2011). Control of material removal of fused silica with single pulses of few optical cycles to sub-picosecond duration. J. Appl. Phys. A.

[49] Melosh H.J. (2007). Hydrocode equation of state for SiO_2_. Meteorit. Planet. Sci..

[50] Medvedev A.B. (2016). Equation of state of silicon dioxide with allowance for evaporation, dissociation, and ionization. Combust. Explos. Shock Waves.

